# Nursing resources and patient outcomes in intensive care units: A protocol for systematic review and meta-analysis

**DOI:** 10.1097/MD.0000000000024507

**Published:** 2021-02-12

**Authors:** Xiaoyan Xu, Haiyan Zhang, Jin Ding, Ying Liu, Jiming Zhang

**Affiliations:** a Department of Critical Care Medicine, Affiliated Hospital of Gansu University of Traditional Chinese Medicine, Lanzhou city; b Department of Critical Care Medicine, Qingyang hospital of Traditional Chinese medicine, Qingyang city; c Department of Nursing; d Department of Emergency; e Department of Internal Medicine, North Hospital, Affiliated Hospital of Gansu University of Traditional Chinese Medicine, Lanzhou city, Gansu province, China.

**Keywords:** intensive care units, nursing, outcomes assessment

## Abstract

**Background:**

As nursing resources is directly related to patient outcomes in the intensive care unit setting, identifying factors related to nursing resources at various levels could contribute to improving those outcomes. This study aims to determine the association of nursing resources with outcomes of intensive care unit patients.

**Method:**

This study adheres to the Preferred Reporting Items for Systematic Reviews and Meta-analysis for Protocols. Chinese electronic Database (Chinese Biomedical Literature Database, Wanfang, and China National Knowledge Infrastructure) and international electronic databases (PubMed, Embase, Cochrane Library, and Web of Science) will be searched for all relevant published articles, with no restrictions on the year of publication or language. Study selection, data collection and assessment of study bias will be conducted independently by a pair of independent reviewers. The Newcastle–Ottawa Scale tool will be used for the risk of bias assessment. The Grading of Recommendations Assessment Development and Evaluation system will be used to assess the quality of evidence. The statistical analysis of this meta-analysis will be calculated by Review manager version 5.3.

**Results:**

The results of this study will be published in a peer-reviewed journal.

**Conclusion:**

The findings of this systematic review will provide a high-quality synthesis of latest evidence and provide a basis for assessing the association of nursing resources on patients’ outcomes in intensive care units.

**Trial registration number:**

10.17605/OSF.IO/9FNEX

## Abstract 2

Supplemental Digital Content is available in the text

## 1 Introduction

Intensive care units (ICUs) were not designed simply to care for the most seriously ill patients, but for those for whom survival was possible, but not certain. During ICU admission, patients experience a variety of physical and psychological stressors, which may result in psychological disorders including anxiety, depression, and post-traumatic stress disorder.^[1,2]^ Over time, these factors can also have detrimental consequences on other health outcomes, thereby affecting the patient's recovery.^[3]^ Considering the ICU as a specialised unit for the care of seriously ill and unstable patients,^[4]^ increasing and maintaining nursing resource, which includes both direct and indirect nursing care, are especially important in the ICU. Furthermore, health care on ICU focuses on the most critically ill patients and consumes a significant portion of medical expenses. Concerns about patient safety and the quality of care are driving research on the clinical and cost-effectiveness of health care interventions, including the rational allocation of nurse resources to maximize their effects.

Nursing resource (e.g., nurse staffing (nurse–patient ratios), nursing performance, nurses’ level of education, training and experience) is an important predictor of patient outcomes in ICU, has a direct influence on infection and mortality rates of patients.^[5]^ Adequate nursing resources is essential for health care quality and safety in ICU patients. Nurses are important members of the ICU team and are responsible for the provision of holistic care to patients.^[6]^ As a 24-hour care provider, nurses act as the hospital's monitoring system for the early detection and prevention of adverse events, they need to assess critically ill patients’ conditions, address nursing problems and perform nursing interventions that directly influence patient outcomes.^[7]^ Patients admitted to intensive care were to be closely observed by skilled nurses capable of intervening clinically and of mobilizing the resources of the hospital on their behalf.^[8]^

Several previous large-scale studies suggest that there may be a link between nursing factors and the development of hospital mortality.^[9,10]^ Recent studies have shown that nurse staffing, nursing performance, nursing work environment, and nurses’ level of experience have a single or composite influence on patients.^[8,11,12]^ For example, nurses’ performance was found to improve with their age and work experience increase.^[13]^ Nurses with higher education tend to make better patient care decisions based on their knowledge from advanced study.^[14]^ Also, some previous SRs have reported on this area.^[8,15]^ Yet evidence to support nursing resource for patient outcomes in ICU remains unclear or inconclusive due to focused on individual factors, low sample sizes and methodological defects.

Considering evaluation of associations with patient outcomes requires integration of multiple aspects of the quality of nursing care, and most previous studies have addressed those factors only individually or in pairs. We will perform a comprehensive systematic review to evaluate the association of nursing resources with outcomes of ICU patients, and identifies where further research is required.

## 2 Materials and methods

### 2.1 Study registration

This protocol will be reported according to preferred reporting items for systematic review and meta-analysis protocols (PRISMA-P).^[16]^ As a part of our project, this study protocol has been registered on the open Science framework (OSF) (Registration DOI: 10.17605/OSF.IO/9FNEX).

### 2.2 Search strategy

We will conduct a systematic search without language restrictions. Four international electronic databases (PubMed, Embase, Cochrane Library, and Web of Science) and three Chinese electronic databases (Chinese Biomedical Literature Databases, Wanfang database, and China National Knowledge Infrastructure) will be searched. The search terms and basic search strategy were as follows: (intensive care units OR “icu” OR “intensive care” OR “critical care” OR “critical illness” OR “emergency medical services”) AND (“nursing staff” OR “nursing resources” OR “hospital staffing” OR “nursing performance” OR “nursing education” OR “nursing training”). In addition, to ensure a comprehensive data collection, we will review the references of included studies, review articles, and conference abstracts. Furthermore, reference lists of the included studies will manually screen for relevance to identify potential studies missed in the systematic search. We did not restrict the study to source, country or publication date and we will provide specific search strategy sample of PubMed and will be shown in Supplemental Digital Content (Appendix 1, http://links.lww.com/MD/F669).

### 2.3 Selection criteria

#### 2.3.1 Types of studies

We will include types of study are observational studies including cross-sectional studies, cohort studies, and case-control studies.

#### 2.3.2 Types of participants

We will include participants who were admitted to any ICU as emergency, medical, or postoperative elective surgical patients, regardless of their status (e.g., conscious, unconscious, intubated), gender, ethnicity or length of stay. We did not limit types of participants by severity of the condition.

#### 2.3.3 Types of outcome measures

We will include the following outcomes. The primary outcome includes the

(1) Mortality,
(2) Length of stay in ICU,
(3) Organ dysfunction as defined by the sequential organ failure assessment (SOFA) score and
(4) Any adverse effects (adverse reactions or events).

The secondary outcomes consist of

(1) Economic outcomes (use for health care, health state utilities, costs of health care, incremental cost-effectiveness cost-effectiveness),
(2) Health-related quality of life (HRQoL) (measured with a validated quality of life questionnaire such as EQ-5D or Short Form-36 (SF-36),
(3) Severity of anxiety and depression in patients (assessed with the Hospital Anxiety and Depression Scale (HADS) or other validated method), and
(4) Patient satisfaction with nursing resources provided (e.g., self-reported).

### 2.4 Exclusion criteria

We will exclude the studies if:

(a) not relevant subject outcome,
(b) the information provided in the results was insufficient for data extraction,
(c) duplicate studies, commentaries, summaries, editorials, letters, or case reports.

### 2.5 Study selection

Two review authors (ZH and XX) will independently screen the titles and abstracts based on the inclusion or exclusion criteria, and discard studies that are not applicable; however, they will initially retain studies and reviews that might include relevant data or information on studies. Two review authors (XX and ZH) will independently assess the retrieved abstracts and, when necessary, the full-text articles to determine which studies satisfy the inclusion criteria. Any discrepancies regarding inclusion will resolved through discussion or by consulting a third member of (ZJ) the review team until consensus is reached. We will record the selection process insufficient detail to complete a PRISMA flow chart.

### 2.6 Data extraction

Two review authors (DJ and LY) will independently extract data from the selected studies using a predefined data extraction form in an Excel spreadsheet. We will resolve any disagreements by discussion and consultation with a third review author (ZJ). When necessary data are unavailable from the study report, we will try to obtain them through correspondence with the study authors.

We will extract from each included study the following information:

(a) basic character of the included research object (author, publication year, study country, the participant numbers in ICU, duration of period studied, source of recruitment);
(b) general demographic characteristics (age, gender, clinical baseline characteristics, nurse-patient ratios, nurses; level of education, training and experience);
(c) outcomes: measures and results of the primary and secondary outcomes (including of mortality, Length of stay in ICU, adverse events, economic outcomes and patient satisfaction) that were reported in the included studies.

If the information present was unclear or if information was missing, the corresponding author of the study will try to contact via email.

### 2.7 Assessment of risk of bias in included studies

Paired reviewers will evaluate independently the risk of bias of included studies using the Newcastle–Ottawa Scale for cross-sectional, cohort, and case-control studies.^[17,18]^ The scale is given a score of 0 to 9 based on selection (4 items), comparability (1 item), and outcome (3 items). We will represent “low,” “medium,” and “high” quality research with scores of 0 to 3, 4 to 6, and 7 to 9, respectively. Any discrepancies in all quality assessments will be resolved after mutual consent and discussion. The scoring results will be presented on a table and we will assess the risk of bias in eligible studies.

### 2.8 Data synthesis and analysis

We will use Cochrane Review Manager (version 5.4.1; The Cochrane Collaboration, Oxford, United Kingdom) software to synthesize the available data. We will pool results from clinically similar studies. If data provided in the study was sufficient and have sufficiently homogenous for analysis, we will present a quantitative analysis. For dichotomous outcomes, we will combine RRs with 95% CIs from included studies. We will report adverse event outcomes narratively if a quantitative analysis is not possible. For continuous outcomes, we will calculate MD, or SMD if studies measure the outcome on different assessment scales, with 95% CIs. If *P* < .05 and I^2^ > 50%, the random effects model was selected to calculate the pooled effective size. In other cases, the fixed-effects model was employed. In addition, if we are unable to perform a meta-analysis due to substantial differences between included studies, we will perform a narrative synthesis of the data.

### 2.9 Assessment of heterogeneity

Heterogeneity among the included studies using the Chi-squared test and the I^2^ statistic. When the I^2^ statistic value is greater than 50% (substantial heterogeneity), we will perform subgroup and sensitivity analyses to consider possible reasons for heterogeneity.

### 2.10 Quality of evidence rating

Two authors will use the Grades of Recommendations, Assessment, Development, and Evaluation (GRADE) approach to assess the quality of evidence and summarize each outcome.^[19,20]^ We will assign 4 categories of evidence quality based on the overall GRADE scores for each comparison:

- high (at least 4 points overall),
- moderate (3 points),
- low (2 points) and
- very low (one point or less).^[21]^

Any disagreement was resolved through discussion and consultation with a third author.

### 2.11 Ethics and dissemination

This study belongs to the category of systematic review and it is only a secondary analysis of the published data, so ethical approval is not applicable to this study.

## 3 Discussion

The nursing resources is a critical factor influencing the quality of patient care in ICU. Nevertheless, demonstrating the impact of high-quality nursing care on quantifiable outcomes to make their contribution visible remains a challenge. Therefore, exploring this question with a review of observational studies remains the best alternative and provides insights for planning future studies. So this review will conduct to investigate the impact of nursing resources on patient outcomes in ICU in the published literature, which will provide clear evidence for clinical workers and nursing managers and improve clinical outcomes of patient in ICU.

There are strengths in this study. First, strict inclusion and exclusion criteria will be employed, including a comprehensive search strategy, which takes into account a wider range of outcome indicators (mortality, adverse effects, length of stay in ICU, economic outcomes, quality of life. etc.). Second, 2 authors will perform independently study selection, data extraction and quality assessment, in order to ensure that all included studies are not personal bias. This shortage of systematic review is due to language barriers, only 2 languages of the trials can be included, other related studies may be missing. Furthermore, the included types of studies are varied, for example cross-sectional studies, case control studies and cohort studies, this may cause substantial heterogeneity.

## Author contributions

**XY Xu:** planned and designed the research

**XY Xu and JM Zhang:** project development, data collection, analysis and interpretation, manuscript writing, article revised

**J Ding:** data collection, analysis and interpretation, manuscript writing

**Y Liu:** data collection, analysis and interpretation

**XY Xu and HY Zhang:** academic oversight and edited all drafts, article revised

**Conceptualization:** Xiaoyan Xu, Haiyan Zhang, Jiming Zhang.

**Data curation:** Haiyan Zhang, Jin Ding.

**Formal analysis:** Jin Ding.

**Investigation:** Jin Ding, Ying Liu.

**Methodology:** Jin Ding, Ying Liu.

**Project administration:** Jiming Zhang.

**Resources:** Ying Liu.

**Software:** Jin Ding, Ying Liu.

**Supervision:** Ying Liu.

**Validation:** Ying Liu.

**Visualization:** Xiaoyan Xu, Haiyan Zhang.

**Writing – original draft:** Xiaoyan Xu, Haiyan Zhang, Jiming Zhang.

**Writing – review & editing:** Xiaoyan Xu, Jiming Zhang.

All authors critically revised the Article for important intellectual content and approved the final version of the manuscript.

## Abbreviations

Abbreviations: GRADE = Grading of Recommendations Assessment Development and Evaluation, ICU = intensive care unit.
